# Engaging with intelligent voice assistants for wellbeing and brand attachment

**DOI:** 10.1057/s41262-023-00321-0

**Published:** 2023-04-05

**Authors:** Catherine Prentice, Sandra Maria Correia Loureiro, João Guerreiro

**Affiliations:** 1 School of Business, University of Southern Queensland, Springfield, QLD Australia; 2 ISCTE – Instituto Universitário de Lisboa, Business Research Unit (BRU-IUL), Lisbon, Portugal

**Keywords:** Intelligence voice assistant, Self-determination, Customer engagement, Wellbeing, Brand attachment

## Abstract

The study draws upon the theories of self-determination and motivation of expectancy to examine how intrinsic motives drive consumers to engage with artificial intelligence (AI) powered intelligent voice assistants (IVAs). The study also explores how consumer engagement leads to their wellbeing and attachment to these AI gadgets and their associated brands. Engagement in this study refers to consumers’ usage and involvement with IVAs. Subject wellbeing was modeled as a mediator between consumer engagement and brand attachment. The research was conducted in the USA with respondents who had owned and used at least one type of IVAs (e.g., Siri, Google Home, Alexa). A range of statistical procedures including structural equation modeling were undertaken to assess the proposed relationships. The results show that the consumer’s need for autonomy, sense of competence, and relatedness are significantly related to IVA engagement. Consumer wellbeing also had a significant mediation effect on the relationship between engagement and brand attachment. The study is the first to link consumer engagement, individual wellbeing, and brand attachment. The study contributes to positive psychology and branding research by integrating mental health and branding effects. The findings have implications for marketing and psychology practitioners.

## Introduction

Intelligent voice assistants (IVAs) are artificial intelligence (AI) powered smart devises that are able to engage in conversations with human beings and perform activities on demand. These smart devices are referred to as conversational agents that can interpret and learn from data to perform tasks (Kaplan and Haenlein 2019). Companies such as Google (Google Home), Amazon (Alexa), and Apple (Homepod, Siri) have launched a series of IVAs over the years. IVAs can be embedded in multiple devices at the same time, for example, in Google Home, Alexa, or augmented reality glasses (Vuzix 2021). These IVAs permeate in households, performing intelligent tasks (e.g., controlling appliances connected to the Internet of Things) and helping users perform daily activities (e.g., schedule meetings or purchase products) (Schweitzer et al. 2019). There were 4.2 billion of IVAs sold globally in 2020 (Statista 2021) with sales varying across different IVA brands and competition between brands (e.g., Google, Apple, Amazon) is intensifying.

Research on smart devices has emerged rapidly given the growing number of IVAs in the market and use in various contexts (e.g., households, work, study). Most studies have focused on identifying the factors related to the usage of smart devices using technology acceptance models such as TAM, TAM2, and TAM3, UTAUT2 (Davis 1989; Venkatesh and Davis 2000; Venkatesh and Bala 2008; Venkatesh et al. 2012; Ashfaq et al. 2020). Few studies have attempted to explore the underlying motives of consumer engagement with IVAs and the subsequent outcomes. McLean and Osei-Frimpong (2019) applied user friendliness to examine consumer engagement with IVAs. No study to date has attempted to understand the intrinsic drivers of consumer engagement and its relationship with IVAs.

Consumer or customer engagement has been extensively discussed in the literature over the past decade (Prentice et al. 2019a, b; Prentice and Nguyen 2020; Prentice et al. 2019a, b; Prentice et al. 2020; Rasool et al. 2020; Santos et al. 2022; Tsai and Men 2017). Research has approached the subject from an individual, organizational, or environmental perspective to examine the drivers of user engagement (e.g., firm initiatives, economic environment, customer characteristics) (Prentice et al. 2019a, b; van Doorn et al. 2010). Very few studies have attempted to understand the intrinsic motivations of consumer engagement. This study draws upon self-determination theory (Deci and Ryan 2012) to examine how consumers are internally driven to engage with IVAs.

Research on consumer engagement is often focused on firm-related outcomes such as purchase and loyalty behaviors (So et al. 2016; Thakur 2016; Prentice et al. 2018a, b; Prentice and Nguyen 2020). No study to date has attempted to understand how consumer engagement may result in individual outcomes for the consumer (e.g., wellbeing). While customer engagement has a range of financial benefits for business, self-determination theory indicates that an inherent reward may be an expected outcome derived from a consumers’ interaction and engagement with a chosen object (Deci and Ryan 2012). The inherent reward for consumers can be their subjective wellbeing, where the IVA is the object. Research (e.g., Shank et al. 2019) has shown that engaging with AI-powered smart devices (e.g., IVAs) generates positive emotions (e.g., happy, amazed). These positive emotions may eventually evolve into consumer attachment to the object-associated brands (Prentice and Loureiro 2018). This discussion leads to the secondary aim of this research to examine how consumer engagement with IVAs affects wellbeing and brand attachment.

The study contributes to customer engagement and artificial intelligence research by understanding the influence of intrinsic motivations on individual wellbeing and brand attachment. The findings may be utilized by IVA manufacturers and the relevant marketers to explore non-organizational initiatives for competitive advantage. The following section presents a review of the relevant literature on the study constructs to form the research hypotheses. The methodology for testing the hypotheses will be outlined, followed by the results of this study. Discussion and implications of these findings will be highlighted for researchers and practitioners.

## Literature review

### Self-determination and consumer engagement

Self-determination theory (Deci and Ryan 2012) indicates that individuals are determined to engage in their choice of behaviors when their needs for autonomy, competence, and relatedness are fulfilled. The need for autonomy refers to a person’s sense of freedom to make their own choices and to not feel constrained or coerced. Competence refers to a person’s tendency to seek a sense of effectiveness, achievement, and challenge in their chosen activities. Relatedness refers to a sense of closeness and connection with others, and a person’s need to be cared for and to avoid feeling isolated. The user has a sense of autonomy when interacting with IVAs as these devices have their own voice, agency skills, and a degree of autonomy. The relationship is often anthropomorphized (Schweitzer et al. 2019). Perceived usefulness and ease of use of IVAs creates a sense of user competence (McLean et al. 2021). IVAs have been personified by some users, as a “nice, friendly, helpful, reliable person with a ready-to-please character, who acts professionally, as well as somewhat subserviently, and remotely” (Schweitzer et al. 2019, p. 703). These characteristics enable users to rely on IVAs to perform daily tasks such as ordering food, placing a call, or controlling appliances. Such dependence ultimately leads to a personalized connection between the user and the IVA and fulfills the user’s sense of relatedness.

Consumer engagement is a multidimensional concept aggregating cognitive, emotional, and behavioral dimensions (e.g., van Doorn et al. 2010; Brodie et al. 2011; Hollebeek et al. 2014) and can be related to a range of business-related outcomes (Prentice et al. 2019a, b; Prentice and Nguyen 2020). Consumer brand engagement is often conceptualized as a psychological state that is distinct to behavioral manifestations (France et al. 2016). Research has tended to approach brand engagement from a firm perspective and has indicated that organizational offerings and marketing promotions affect consumer brand engagement (De Vries and Carlson 2014; Powell 2016; Whelan and Wohlfeil 2006). However, the concept of engagement is often reflective of an interactive connection with an object (Brodie et al. 2011). The object can be a human employee or a tangible object such as an IVA. In this study, consumer—IVA interactions refer to consumer’s behavioral, cognitive, and affective engagement with the digital assistant. The level of connection can be determined by the different degrees of autonomy, relatedness, and competence (Loroz and Braig 2015; Thomson 2006).

Research (McLean et al. 2021) shows that IVAs’ physical attributes (social presence, perceived intelligence, social attraction), technology attributes (perceived ease of use, perceived usefulness), and situational attributes (utilitarian benefits and distrust, a negative relationship) may motivate consumers to interact with IVAs and engage with the associated brands. The level of engagement depends on the degree of autonomy (the consumer’s feeling of control or being controlled by the IVA), the degree of relatedness (how strong is the human self-connection with the IVA), and the consumers competence (the degree of the utilitarian benefits extracted from the relationship). Thus, the following hypothesis is offered:

#### H1

The need for autonomy is positively related to consumer engagement with an IVA.

#### H2

The need for competence is positively related to consumer engagement with an IVA.

#### H3

The need for relatedness is positively related to consumer engagement with an IVA.

### Consumer engagement and wellbeing

Wellbeing is a complex phenomenon involving internal and external mechanisms, and generally includes subjective wellbeing (SWB) and authentic happiness (Christopher and Hickinbottom 2008). SWB reflects the achievement of contentment or satisfaction that is subjective and inherent in human beings (Diener et al. 2003). Authentic happiness emerges from positive emotions, a sense of connection and purpose to life, and through achievements and enriching connections. SWB is an embedded internal mechanism, whereas authentic happiness is an external mechanism. Internally, humans develop subjective mental perceptions by focusing on a particular event and activating emotional regulation. The internal mechanism requires persistence and resilience (Lutz et al. 2013). The external mechanism, however, is more circumstantial and dependent on external events (Hausman and McPherson 2006) (e.g., the interaction with an IVA), reflects emotions (Van Boven and Gilovich 2003), and has a sense of meaning and personal fulfillment (Bhattacharjee and Mogilner 2014). The current study conceptualizes authentic happiness as user wellbeing resulting from interactions with an IVA.

Research on the consequences of customer engagement has generally focused on business- or company-related benefits (e.g., Pansari and Kumar 2017; Prentice et al. 2019a, b, 2018a). Little research has focused on the personal benefits for consumers or customers (Prentice and Loureiro 2018). However, the expectancy theory of motivation (Vroom et al. 2015) explains how consumers may engage with a brand or product for their own benefit. The theory suggests that individuals engage in a certain behavior to achieve a desired outcome. The outcome may be financial or psychological, such as wellbeing. Research has shown that engaging with AI-powered robots may bring users pleasure (e.g., as a companion for older adults, sex robots) (Chen 2018; Fosch-Vilaronga and Poulsen 2020; Moyle et al. 2013). IVAs can also provide services on demand such as finding entertainment for users without the need for compensation or reciprocation. The service nature provides users pleasure and entertainment which affects wellbeing. The process of interacting with IVAs (e.g., conversing with Siri, asking Google home for the time, playing Frank Sinatra) is reflective of user engagement with the IVA. Thus, the following hypothesis is offered:

#### H4

Consumer engagement with an IVA has a positive influence on wellbeing.

### Consumer engagement and brand attachment

Attachment has been defined as “a multi-faceted property of the relationship between a specific individual or group of individuals and a specific material object and/or specific individual or group of individuals that includes a deep psychological and emotional connection” (Saldanha et al. 2020, p. 436). Attachment theory is grounded in a motivational–behavioral control system as a response to caregiving figures and can be integrated into the personality structure to explain personal attachment (Bowlby 1969). This theory can explain the relationship between consumers and brands (Saldanha et al. 2020; Park et al. 2013; Fournier 1998) and the patterns of engagement with an object (Mikulincer and Shaver 2007). When a person is comfortable and intimate with an object (e.g., IVA), the discovery system (Bowlby 1973) is activated. This activation can augment engagement with the object and help to develop a sense of attachment to the object and its associated entities. A consumer’s engagement with an IVA may lead to attachment with the IVA and its associated brand (e.g., Siri–Apple, Google Home–Google). Thus, the following hypothesis is offered:

#### H5

Consumer engagement with an IVA is positively related to IVA attachment.

### Consumer engagement, wellbeing, and attachment

Attachment theory can be used to explain consumer brand relationships (Fournier 1998). The theory (Bowlby 1980) offers that the degree of emotional attachment to a peer, or an object, is an indicator of the nature of interaction and engagement. Similar to how humans establish a bond with friends or relations, consumers tend to develop a close relationship with an object or a brand. Some examples include brand—celebrity relationships (O'Guinn 1991; Thomson 2006), product brands (Park et al. 2013; Thomson et al. 2005), and place brands (Debenedetti et al. 2014). Stronger attachment often results from stronger feelings and affection (e.g., Sternberg 1987; Aron and Westbay 1996; Thomson et al. 2005), such as happiness. Engaging with an IVA can result in consumer satisfaction or happiness and may result in attachment to the IVA (e.g., Hwang and Lyu 2015; Nicolao et al. 2009). Thus, the following hypothesis is offered:

#### H6

Subjective wellbeing has a positive influence on IVA attachment.

Engagement can be an emotional gratification experience taking place consciously or unconsciously (Bartsch et al. 2008), for example, entertainment experiences (Rubin 1983). When engaging with an IVA, the relationship between the person and the object is stablished. The attention drawn from the engagement may lead to a sensation of gratification when the engagement experience is meaningful and fulfilling. Gradually, the user may develop a sense of attachment to the IVA. Thus, the following hypothesis is offered:

#### H7

Subjective wellbeing has a significant mediation effect between consumer engagement and IVA attachment.

## Methodology

### Sampling

The data were collected in the USA in February 2021 utilizing Amazon’s Mechanical Turk (MTurk), which is considered to be a cost-effective and reliable source (Buhrmester et al. 2016; Paolacci et al. 2010). To ensure the data quality, quality assurance measures (attention checks, spending traps) were adopted. The study targeted respondents who had used at least one IVA for more than a year to understand the impact of engagement with the IVA. Screening questions filtered those who did not meet the selection criteria. To encourage participation, each respondent was compensated with USD 1.00 for completing the questionnaire. To minimize response fatigue, the questionnaire was designed to ensure a short completion time of less than 10 min.

### Measurement items

The items used to measure the constructs of autonomy, competence, relatedness, and attachment were adapted from Thomson (2006). Each item was reworded to reflect their relevance to IVAs. The items used to measure customer engagement were adapted from So et al.’s (2016) multidimensional customer engagement scale, reflecting users’ affective, cognitive, and psychological engagement with a brand. The measure consists of five dimensions including identification, absorption, attention, enthusiasm, and interaction. This scale has proven reliability and validity and has been widely used in brand engagement studies (e.g., Li et al. 2020; Petzer and Tonder 2019; Rasoolimanesh et al. 2019). Subjective wellbeing was measured by adapting measures from Van Boven and Gilovich (2003) and Bhattacharjee and Mogilner, (2014) to reflect the authentic happiness as a result of interaction and engagement with IVAs. A 7-point Likert scale (1—strongly disagree to 7—strongly agree) was used to measure the items. Given that these measures were adapted from existing studies, reliability and validity were assessed by confirmatory factor analysis.

### Data collection procedure

The questionnaire was developed to minimize recall and common method bias through the employment of memory message (e.g., think about the IVA you have, please answer the questions), commitment reinforcement (e.g., please answer conscientiously and anonymously), and attention (e.g., what is the color of the sea? Make sure to select pink to let us now that you are paying attention). The items of the same construct were physically distanced, and terms were straightforward to avoid confusion. The measurement items for the different dimensions of a construct were spread throughout the questionnaire and negative wording was used to ensure consistency.

Prior to conducting the survey, a pilot study was conducted with 15 randomly chosen consumers who had used IVAs to ensure appropriate wording and completion time. Minor revisions were made as a result of the pilot study. The survey was designed to ensure anonymity. Completion of the online survey was taken as an indication of the respondent’s willingness to participate in the study (Prentice and Nguyen 2020).

A total of 259 responses were received after two weeks. After excluding those with missing values and outliers, 222 remained for further analysis. Almost half of the respondents (48%) had used Amazon Alexa, followed by Apple Siri (28%), and Google Assistant (17%). Almost half of respondents (46.8%) had owned their IVAs for more than two years. Respondents reported various usage with 37.4% using the IVAs on a daily basis, 24.8% 2 to 3 times a week, and 6% used the IVA once a month. The majority (52%) used IVAs more often during 2021 due to the COVID-19 pandemic. Table 1 presents the sociodemographic profiles of the participants.

**Table 1:** Sociodemographic and details of the participants

Variables	Frequency	Percentage (%)
*Gender*		
Female	114	51.4
Male	107	48.2
Other	1	0.5
*Age*		
18–24	20	9.0
25–34	97	43.7
35–44	50	22.5
45–54	28	12.6
55–64	20	9.0
>65	07	3.2
*Education level*		
Less than high school	1	0.5
High school graduate	15	6.8
Some college	51	23.0
Professional degree	17	7.7
College graduate	93	41.9
Master’s degree	43	19.4
Doctorate	2	0.9
*Technology expertise*		
Very experienced	54	24.3
Experienced	95	42.8
Average user	69	31.1
Not experienced	4	1.8
*Household size*		
1–2 Persons	89	40.1
3 Persons	60	27.0
Above 3 persons	73	32.9

### Common method bias

In addition to the previously stated ex-ante procedures to minimize common method bias (CMB), ex-post statistical remedies were also conducted to assess CMB. Harman’s single factor test, partial correlation procedure, and controlling for the effects of an unmeasured latent method factor were assessed. For the Harman’s single factor test, all measurement items were loaded on one factor. The *R*^2^ values demonstrated that the model explained 71.6% of the variance in attachment strength, 72.8% of the variance in human–IVA engagement, and 75.1% of the variance in happiness. Predictive validity was ensured by the positive values of Q^2^, calculated with the blindfolding procedure of the Stone–Geisser test. A marker (the attitude toward the color blue) was also included to analyze common method variance (CMV). Consistent with the approach in Chin et al. (2013), the marker variable (Table 6) was added as an exogenous variable to regress against other endogenous variables. All significant parameter estimates in the model were equal or slightly smaller in the marker variable model. These results indicate that CMB was not an issue in this study.

## Analysis and results

### Measurement model

The model was tested using Smart PLS 3.0 and the repeated indicators approach for the second-order reflective-reflective construct (user engagement). The option for PLS was determined by its merit of being able to estimate complex models with many variables, indicators, and structural paths, without imposing distributional assumptions on the data. The outer model was assessed through the constructs’ reliability and validity. Cronbach’s alpha (CA) and composite reliability (CR) scores demonstrate the constructs’ reliability (Table 2).

**Table 2:** Measurement results

Construct	FL	CA	rho_A	CR	AVE
*Autonomy*		0.821	0.821	0.918	0.848
A1. IVA makes me feel controlled (reversed)	0.921				
A2. IVA makes me feel pressured to be certain ways (reversed)	0.921				
A3. IVA makes me feel free to be who I am					
*Competence*		0.908	0.909	0.956	0.915
C1. Generally. IVA makes me feel very capable	0.959				
C2. Generally. IVA makes me feel effective	0.955				
C3. IVA makes me feel inadequate or incompetent (reversed)					
*Relatedness*		0.922	0.925	0.962	0.928
R1. IVA makes me feel cared about	0.961				
R2. I feel a lot of closeness with IVA	0.966				
*Attachment strength*		0.919	0.920	0.943	0.805
AT1. I feel better if I am not away from or without IVA for long periods of time	0.863				
AT2. I miss IVA when I don’t have it with me	0.908				
AT3. If IVA was permanently gone from my life. I would d be upset	0.912				
AT4. Losing IVA forever would be distressing to me	0.906				
*Absorption*		0.956	0.957	0.965	0.821
AB1. When I am interacting with this IVA. I forget everything else around me	0.910				
AB2. Time flies when I am interacting with this IVA	0.937				
AB3. When I am interacting with this IVA I get carried away	0.924				
AB4. When interacting with this IVA. It is difficult to detach myself	0.878				
AB5. In my interaction with this IVA. I am immersed	0.931				
AB6. When interacting with this IVA intensely. I feel happy	0.855				
*Attention*		0.940	0.945	0.954	0.805
ATT1. I like to learn more about this IVA	0.875				
ATT2. I pay a lot of attention to anything about this IVA	0.926				
ATT3. Anything related to this IVA grabs my attention	0.922				
ATT4. I concentrate a lot on this IVA	0.893				
ATT5. I like learning more about this IVA	0.869				
*Enthusiasm*		0.949	0.951	0.961	0.832
E1. I am heavily into this IVA	0.906				
E2. I am passionate about this IVA	0.927				
E3. I am enthusiastic about this IVA	0.914				
E4. I feel excited about this IVA	0.898				
E5. I love this IVA	0.906				
*Identification*		0.956	0.957	0.968	0.884
I1. When someone criticizes this IVA. It feels like a personal insult	0.912				
I2. When I talk about this IVA. I usually say “we” rather than “they”	0.949				
I3. This IVA’s successes are my successes	0.935				
I4. When someone praises this IVA. It feels like a personal compliment	0.963				
*Interaction*		0.972	0.972	0.978	0.901
IN1. In general. I like to get involved in the IVA's community discussions	0.928				
IN2. I am someone who enjoys interacting with like-minded others in the IVA's community	0.959				
IN3. I am someone who likes actively participating in the IVA's community discussions	0.951				
IN4. In general. I thoroughly enjoy exchanging ideas with other people in the IVA's community	0.964				
IN5. I often participate in activities of the IVA's community	0.943				
*Wellbeing*		0.930	0.931	0.955	0.877
HA1. The experience with IVA contributed very much to my happiness in life	0.921				
HA2. The experience with IVA is very meaningful	0.946				
HA3. The experience with IVA is very personally fulfilling	0.943				

FL = Factor loading; CA = Cronbach’s alpha; CR = composite reliability; d = deleted; AVE—average variance extracted; a—item eliminated

Discriminant validity was assessed by two criteria, the Fornell and Larcker criterion and the Heterotrait/Monotrait ratio. The Fornell and Larcker criterion indicated that the squared AVE values should be higher than the inter-correlation values (Fornell and Larcker, 1981). Values of AVE higher than 0.5 revealed that the constructs had convergent validity. The Heterotrait/Monotrait ratio should be lower than 0.9. The scores of VIF (variance inflation factor) were below 3.33, demonstrating that construct attachment strength did not pose inner collinearity issues (Diamantopoulos and Siguaw, 2006). These results are presented in Tables 3, 4, and 5.

**Table 3:** Discriminant validity—Fornell and Larcker’s criterion

Variables	1	2	3	4	5	6	7	8	9	10
1.Attachment strength	0.897									
2.Autonomy	− 0.565	0.921								
3.Competence	0.606	− 0.305	0.957							
4.Happiness	0.763	− 0.543	0.638	0.936						
5.Relatedness	0.830	− 0.567	0.627	0.780	0.963					
6.absorption	0.769	− 0.654	0.585	0.823	0.815	0.906				
7.attention	0.809	− 0.479	0.665	0.807	0.794	0.810	0.897			
8.enthusiasm	0.831	− 0.495	0.657	0.809	0.803	0.815	0.895	0.912		
9.identification	0.782	− 0.675	0.474	0.760	0.778	0.854	0.751	0.797	0.940	
10.interaction	0.768	− 0.607	0.557	0.798	0.814	0.866	0.810	0.785	0.822	0.949

The diagonal refers to the squared AVE values. The lower diagonal refers to the inter-correlation values

**Table 4:** Discriminant validity—HTMT ratio

Variables	1	2	3	4	5	6	7	8	9	10
1.Attachment strength										
2.Autonomy	0.650									
3.Competence	0.662	0.353								
4.Happyness	0.824	0.621	0.694							
5.Relatedness	0.900	0.652	0.686	0.843						
6.Absorption	0.820	0.739	0.628	0.872	0.867					
7.Attention	0.865	0.537	0.723	0.858	0.846	0.847				
8.Enthusiasm	0.887	0.558	0.709	0.860	0.855	0.853	0.843			
9.Identification	0.834	0.763	0.508	0.805	0.828	0.893	0.783	0.833		
10.Interaction	0.812	0.680	0.593	0.838	0.860	0.898	0.840	0.814	0.852	

HTMT refers to Heterotrait–Monotrait ratio

**Table 5:** Collinearity assessment for structural model

Variables	Attachment strength	Autonomy	Competence	Human–IVA engagement	Happiness	Relatedness	Absorption	Attention	Enthusiasm	Identification	Interaction
Attachment				1.000	3.171						
Autonomy	1.483										
Competence	1.660										
User engagement					3.171		1.000	1.000	1.000	1.000	1.000
User wellbeing											
Relatedness	2.219										

VIF (Variance inflation factor) < 3.3; Dependent variable: attachment strength

### Hypotheses testing

Studies have highlighted the relevance of demographic variables on consumer attitude and behavior. Demographic variables, such as age, gender, household size, and educational level, can influence the IVA-human engagement process and were applied as control variables. The results show that the control variables did not significantly affect the control variables.

The testing supported all hypotheses, except the relationship between subjective wellbeing and attachment. Autonomy, competence, and relatedness were associated with consumer engagement (*β* = 0.221, *p* < 0.0005; *β* = 0.170, *p* < 0.0005; *β* = 0.663, *p* < 0.0005) and consumer engagement was positively associated with welling and attachment (*β* = 0.784, *p* < 0.0005; *β* = 0.853, *p* < 0.0005) (Table 6).

**Table 6:** Results of structural analysis

Direct effects for the proposed relationships	Model 1	Model 2	Model 3	Model 4	
Autonomy → engagement	0.221***				
Competence → engagement	0.170***				
Relatedness → engagement	0.633***				
User engagement → welling		0.784***			
User engagement → attachment			0.853***		
Wellbeing → attachment				0.095	

	*R*^2^	*Q*^2^ (= 1-SSE/SSO)			
User engagement	0.728	0.521			
User wellbeing	0.751	0.650			
Attachment	0.716	0.568			

Indirect effects (mediation)	PC	T statistics (|O/STDEV|)	*P* value	Bias corrected confidence interval	
				Lower bound	Upper bound
Autonomy → engagement → attachment	− 0.129	2.531	0.012*	− 0.223	− 0.038
Competence → engagement → attachment	0.132	3.391	0.001**	0.059	0.209
Relatedness → engagement → attachment	0.552	10.647	0.000***	0.452	0.654
Engagement → wellbeing → attachment	0.669	10.924	0.000***	0.546	0.791
Autonomy → engagement → wellbeing → attachment	− 0.101	2.461	0.014*	− 0.184	− 0.028
Competence → engagement → wellbeing → attachment	0.104	3.234	0.001**	0.047	0.169
Relatedness → engagement → wellbeing → attachment	0.433	7.573	0.000***	0.337	0.554

***p < 0.001; **p < 0.01; *p < 0.05; ns-not significant; VAF: variance accounted for. SRMR = 0.066, rms = 0.176

The bias corrected bootstrapping p value was employed to test the mediating effect. Table 6 shows the bias corrected bootstrapping p values and the path coefficient estimates as the magnitude of the direct and indirect effects. The mediation testing suggests that all indirect effects were significant indicating partial mediation relationships ranging between 0 and 100%, VAF higher than 80% implies full mediation.

## Discussion and implications

IVAs are increasingly being adopted into consumers’ daily lives. These AI-powered smart devices are used as tools for convenience and may provide companionship that does not require compensation or reciprocation. These attributes can result in positive outcomes for users. From a psychological and marketing perspective, the study examined how human–IVA interactions affect consumers’ subjective wellbeing and brand attachment.

The theories of self-determination and expectancy of motivation were employed to propose that the need for autonomy, competence, and relatedness when interacting with IVAs may lead to user happiness and attachment to these gadgets. The results from hypotheses testing confirmed most of the proposed relationships, with consumer engagement and wellbeing having a significant mediating effect on the relationship between the intrinsic motivators and brand attachment.

The significant relationship between self-determination and consumer engagement in this study is consistent with research that found that intrinsic factors drive engagement (Froiland and Worrell 2016; Bhuvanaiah and Raya 2015). The wellbeing–engagement link is also consistent with Rothmann (2008), although the latter is in the organizational context centering on employee wellbeing and engagement. Findings related to consumer engagement and IVA attachment indicate that engaging with a digital assistant may have implications for brand attachment (Ou et al. 2020). Consequently, this study has theoretical and practical implications.

### Theoretical implications

The study contributes to customer engagement research by identifying the intrinsic factors that are not initiated by the brand firm to engage customers. Research has primarily used an organizational initiatives approach such as premium service offering and brand experience to identify the antecedents of customer engagement (e.g., France et al. 2016; Prentice et al. 2019a, b; Roy et al. 2018). This study responds to Prentice et al. (2018a, 2018b) suggestion that an organic approach to consumer engagement should be explored. This approach is focused on identifying personal factors rather than organizational offerings to address customer relationships with the brand and associated products. This study examined how self-determination as a personal factor exerts significant influence on brand engagement and attachment. The findings of this study provide a new stream of research from a non-organizational perspective to understand the motives of customer engagement. In particular, this study broadens the concept of consumer engagement to embrace the aspect of affective and cognitive connection with a personal object and how such connection is elevated to brand attachment. This initiative has far reaching implications for brand engagement research.

This study was the first to examine how personal benefits induce consumer engagement with a product and its associated brand. The findings are consistent with Prentice et al. (2019a, b) and Prentice and Loureiro (2018). These studies addressed consumer engagement from the individual perspectives of self-identity and personal attributes. The study contributes to branding research by exploring how self-centric benefits (subject wellbeing) relate to customer engagement and brand attachment. Customer engagement can result in business-related outcomes such as customer purchase and loyalty (Thakur 2016). This finding presents a new avenue for branding research to explore the intrinsic drivers of brand engagement.

The finding that customer engagement has a significant effect on personal wellbeing has implications for positive psychology research. Studies have mainly approached wellbeing from social, health, and psychological perspectives (e.g., Balaguer et al. 2017). The current study demonstrates that marketing initiatives may affect consumer wellbeing. The factors that influence customer engagement may also affect subjective wellbeing. Research on AI-powered tools has tended to focus on technological acceptance and user friendliness. This study contributes to artificial intelligence research by utilizing self-determination and expectancy theories to reveal how IVAs can be extended from the perspective of technology acceptance to consumer behavior and brand research. This study showcases a broader research stream on AI through the utilization of positive psychology and branding literature.

### Practical implications

Marketing practitioners, IVA manufacturers, and psychologists may benefit from the findings of this study. The significant relationship between intrinsic motives and customer engagement suggests that marketers may not always need to approach to customers/consumers through expensive marketing promotions and other organizational offerings. As shown in this study, consumers are self-motivated to engage with the brands of their choice. Marketers who can identify these intrinsic factors of brand engagement can save substantial costs related to marketing. IVA manufactures and marketers that work collaboratively to identify the attributes associated with IVAs can entice consumers to engage with the brand.

The findings also reinforce the importance of human–AI interaction. Despite the popularity of IVAs, their features and functionality are rather limited and still in its infancy (The Verge 2021). Amazon has only recently launched a male voice for Alexa. IVAs also only respond to humans on demand in a servant type of relationship (Schweitzer et al. 2019). The manufactures that advance technologies that may be able to suggest products and services without prompting may enhance the use of IVAs and encourage consumers’ emotional attachment.

This study shows that human–AI engagement can lead to user positive subjective wellbeing. The finding may help IVA manufactures and marketers to understand that AI agents can go beyond simple functional assistants to enhance sustainable and happy exchanges in the future. Manufactures and marketers who work together to develop AI agents may be able to engage in dialogue with consumers to create personal bonds and enhance user satisfaction. For example, IoT-enabled appliances such as fridges, washing machines, and other functionalities capture daily routines. AI agents can be advanced to offer more personalized experiences.

While research in psychology has attempted to identify the social and psychological factors of subjective wellbeing, this study provides a new perspective on the intrinsic drivers of personal happiness. The study does this by broadening the stereotypical antecedents of individual wellbeing. Practitioners may be able to explore novel therapies for depression or other similar conditions.

## Limitations and future research directions

The study acknowledges limitations that may benefit from further research. First, the current study was conducted in the USA where there is high market penetration for smart devices (Statista 2020). However, the findings may not be applicable in other contexts. The data collected from MTurk may be not representative of all IVA users. Attempts to procure other data sources are recommended. A cross-cultural study may also generate more applicable findings and reveal the role of cultural difference in the use of smart assistants. The study also focused on the evaluation of user wellbeing resulting from IVAs. Wellbeing is known to be dependent on dynamic factors that change with time (Lutz et al. 2013). Therefore, a longitudinal study could explore the effect of engagement on happiness over time. Future research could also explore moderators such as intimacy, level of interdependence, or level of empathy effects between user engagement and wellbeing.

## Conclusion

The study draws on the theories of self-determination and expectancy of motivation and proposes that intrinsic factors may drive consumers to engage with IVAs. It further identifies how engagement may affect wellbeing and attachment. The study was undertaken with consumers in the USA who had utilized at least one IVA brand and the results confirmed the proposed relationships. The findings present a range of theoretical and practical implications. Limitations and future research derived from this study conclude the work.

## References

[CR1] Aron A, Westbay L (1996). Dimensions of the prototype of love. Journal of Personality and Social Psychology.

[CR2] Ashfaq M, Yun J, Yu S, Loureiro SMC (2020). Modeling the determinants of users’ satisfaction and continuance intention of text-based conversational agents. Telematics and Informatics.

[CR3] Balaguer I, Duda JL, Castillo I (2017). Motivational antecedents of well-being and health related behaviors in adolescents. Journal of Human Kinetics.

[CR4] Bartsch A, Vorderer P, Mangold R, Viehoff R (2008). Appraisal of emotions in media use: Toward a process model of meta-emotion and emotion regulation. Media Psychology.

[CR5] Bhattacharjee A, Mogilner C (2014). Happiness from ordinary and extraordinary experiences. Journal of Consumer Research.

[CR6] Bhuvanaiah T, Raya RP (2015). Mechanism of improved performance: Intrinsic motivation and employee engagement. SCMS Journal of Indian Management.

[CR7] Bowlby J (1969). Attachment and loss.

[CR8] Bowlby, J. 1973. *Attachment and loss*, Vol. 2. *Separation: Anxiety and anger*. New York: Basic Books.

[CR9] Bowlby, J. 1980. *Attachment and loss*, Vol. 3. *Loss: Sadness and depression*. New York: Basic Books.

[CR10] Brodie RJ, Hollebeek LD, Jurić B, Ilić A (2011). Customer engagement: Conceptual domain, fundamental propositions, and implications for research. Journal of Service Research.

[CR11] Buhrmester M, Kwang T, Gosling SD, Kazdin AE (2016). Amazon's mechanical Turk: A new source of inexpensive, yet high-quality data?. Methodological issues and strategies in clinical research.

[CR12] Chen, N. 2018, July. Acceptance of social robots by aging users: Towards a pleasure-oriented view. In *International conference on cross-cultural design* (pp. 387–397). Springer, Cham.

[CR13] Chin, Wynne W., Jason B. Thatcher, Ryan T. Wright, and Doug Steel. 2013. Controlling for common method variance in PLS analysis: the measured latent marker variable approach. In *New perspectives in partial least squares and related methods* (pp. 231–239). New York: Springer.

[CR14] Christopher JC, Hickinbottom S (2008). Positive psychology, ethnocentrism, and the disguised ideology of individualism. Theory and Psychology.

[CR15] Davis FD (1989). Perceived usefulness, perceived ease of use, and user acceptance of information technology. MIS Quarterly.

[CR16] De Vries NJ, Carlson J (2014). Examining the drivers and brand performance implications of customer engagement with brands in the social media environment. Journal of Brand Management.

[CR17] Debenedetti A, Oppewal H, Arsel Z (2014). Place attachment in commercial settings: A gift economy perspective. Journal of Consumer Research.

[CR18] Deci, E. L., and Ryan, R. M. (2012). Self-determination theory in health care and its relations to motivational interviewing: a few comments. *International Journal of* 10.1186/1479-5868-9-24 PMC3312850 22385839

[CR100] Diener, E., Oishi, S., and Lucas, R. E. (2003). Personality, culture, and subjective well-being: Emotional and cognitive evaluations of life. *Annual review of psychology* 54(1): 403–425. 10.1146/annurev.psych.54.101601.145056 12172000

[CR19] Fosch-Villaronga E, Poulsen A (2020). Sex care robots. Paladyn, Journal of Behavioral Robotics.

[CR20] Fournier S (1998). Consumers and their brands: Developing relationship theory in consumer research. Journal of Consumer Research.

[CR21] France C, Merrilees B, Miller D (2016). An integrated model of customer-brand engagement: Drivers and consequences. Journal of Brand Management.

[CR22] Froiland JM, Worrell FC (2016). Intrinsic motivation, learning goals, engagement, and achievement in a diverse high school. Psychology in the Schools.

[CR23] Hausman DM, McPherson MS (2006). Economic analysis, moral philosophy, and public policy.

[CR24] Hollebeek LD, Glynn MS, Brodie RJ (2014). Consumer brand engagement in social media: Conceptualization, scale development and validation. Journal of Interactive Marketing.

[CR25] Hwang J, Lyu SO (2015). The antecedents and consequences of well-being perception: An application of the experience economy to golf tournament tourists. Journal of Destination Marketing and Management.

[CR26] Kaplan A, Haenlein M (2019). Rulers of the world, unite! The challenges and opportunities of artificial intelligence. Business Horizons.

[CR27] Li MW, Teng HY, Chen CY (2020). Unlocking the customer engagement-brand loyalty relationship in tourism social media: The roles of brand attachment and customer trust. Journal of Hospitality and Tourism Management.

[CR28] Loroz PS, Braig BM (2015). Consumer attachments to human brands: The “Oprah effect”. Psychology and Marketing.

[CR29] Lutz A, McFarlin D, Perlman DM, Salomons V, Davidson RJ (2013). Altered anterior insula activation during anticipation and experience of painful stimuli in expert meditators. NeuroImage.

[CR30] McLean G, Osei-Frimpong K (2019). Hey Alexa… examine the variables influencing the use of artificial intelligent in-home voice assistants. Computers in Human Behavior.

[CR31] McLean G, Osei-Frimpong K, Barhorst J (2021). Alexa, do voice assistants influence consumer brand engagement?–Examining the role of AI powered voice assistants in influencing consumer brand engagement. Journal of Business Research.

[CR32] Mikulincer M, Shaver PR, Pereg D (2003). Attachment theory and affect regulation: The dynamics, development, and cognitive consequences of attachment-related strategies. Motivation and Emotion.

[CR33] Moyle W, Cooke M, Beattie E, Jones C, Klein B, Cook G, Gray C (2013). Exploring the effect of companion robots on emotional expression in older adults with dementia: A pilot randomized controlled trial. Journal of Gerontological Nursing.

[CR34] Nicolao L, Irwin JR, Goodman JK (2009). Happiness for sale: Do experiential purchases make consumers happier than material purchases?. Journal of Consumer Research.

[CR35] O’Guinn T, Belk Russell W (1991). Touching greatness: The central Midwest Barry Manilow fan club. Highways and buyways: Naturalistic research from the consumer behavior odyssey.

[CR36] Ou J, Wong IA, Prentice C, Liu MT (2020). Customer engagement and its outcomes: The cross-level effect of service environment and brand equity. Journal of Hospitality and Tourism Research.

[CR37] Pansari A, Kumar V (2017). Customer engagement: The construct, antecedents, and consequences. Journal of the Academy of Marketing Science.

[CR38] Paolacci G, Chandler J, Ipeirotis PG (2010). Running experiments on amazon mechanical turk. Judgment and Decision Making.

[CR39] Park CW, Eisingerich AB, Park JW (2013). Attachment–aversion (AA) model of customer–brand relationships. Journal of Consumer Psychology.

[CR40] Petzer, D. J., and Van Tonder, E. 2019. Loyalty intentions and selected relationship quality constructs: The mediating effect of customer engagement. *International Journal of Quality and Reliability Management*.

[CR41] Powell SM (2016). Journal of brand management–year end review 2016. Journal of Brand Management.

[CR42] Prentice C, Loureiro SMC (2018). Consumer-based approach to customer engagement–The case of luxury brands. Journal of Retailing and Consumer Services.

[CR43] Prentice C, Nguyen M (2020). Engaging and retaining customers with AI and employee service. Journal of Retailing and Consumer Services.

[CR45] Prentice C, Han XY, Hua LL, Hu L (2019). The influence of identity-driven customer engagement on purchase intention. Journal of Retailing and Consumer Services.

[CR46] Prentice, C., Wang, X., & Lin, X. 2018a. An organic approach to customer engagement and loyalty. *Journal of Computer Information Systems*. 326–336.

[CR47] Prentice C, Wang X, Loureiro SMC (2019). The influence of brand experience and service quality on customer engagement. Journal of Retailing and Consumer Services.

[CR48] Prentice, C., Wang, X., and Lin, X. 2018b. An organic approach to customer engagement and loyalty. *Journal of Computer Information Systems*.

[CR49] Prentice C, Weaven S, Wong IA (2020). Linking AI quality performance and customer engagement: The moderating effect of AI preference. International Journal of Hospitality Management.

[CR50] Rasool A, Shah FA, Islam JU (2020). Customer engagement in the digital age: A review and research agenda. Current Opinion in Psychology.

[CR51] Rasoolimanesh SM, Md Noor S, Schuberth F, Jaafar M (2019). Investigating the effects of tourist engagement on satisfaction and loyalty. The Service Industries Journal.

[CR52] Rothmann S (2008). Job satisfaction, occupational stress, burnout and work engagement as components of work-related wellbeing. SA Journal of Industrial Psychology.

[CR53] Roy SK, Shekhar V, Lassar WM, Chen T (2018). Customer engagement behaviors: The role of service convenience, fairness and quality. Journal of Retailing and Consumer Services.

[CR54] Rubin AM (1983). Television uses and gratifications: The interactions of viewing patterns and motivations. Journal of Broadcasting.

[CR55] Saldanha N, Mulye R, Rahman K (2020). A strategic view of celebrity endorsements through the attachment lens. Journal of Strategic Marketing.

[CR56] Santos ZR, Cheung CM, Coelho PS, Rita P (2022). Consumer engagement in social media brand communities: A literature review. International Journal of Information Management.

[CR57] Schweitzer F, Belk R, Jordan W, Ortner M (2019). Servant, friend or master? The relationships users build with voice-controlled smart devices. Journal of Marketing Management.

[CR58] Shank DB, Graves C, Gott A, Gamez P, Rodriguez S (2019). Feeling our way to machine minds: People's emotions when perceiving mind in artificial intelligence. Computers in Human Behavior.

[CR60] So KKF, King C, Sparks BA, Wang Y (2016). The role of customer engagement in building consumer loyalty to tourism brands. Journal of Travel Research.

[CR61] Statista 2020. Global smart speaker ownership by country. Retrieved September 30, 2021, from https://www.statista.com/statistics/1130208/smart-speaker-ownership-worldwide-by-country/

[CR62] Statista 2021. Number of Voice Assistants in use Worldwide. Retrieved September 30, 2021, from https://www.statista.com/statistics/973815/worldwide-digital-voice-assistant-in-use/

[CR63] Sternberg RJ (1987). Liking versus loving: A comparative evaluation of theories. Psychological Bulletin.

[CR64] Thakur R (2016). Understanding customer engagement and loyalty: A case of mobile devices for shopping. Journal of Retailing and Consumer Services.

[CR65] The Verge 2021. Alexa finally gets a masculine-sounding voice option. Retrieved September 30, 2021, from https://www.theverge.com/2021/7/21/22587130/alexa-masculine-voice-gender-ziggy-amazon-smart-assistant

[CR66] Thomson M (2006). Human brands: Investigating antecedents to consumers’ strong attachments to celebrities. Journal of Marketing.

[CR67] Thomson M, MacInnis DJ, Park CW (2005). The ties that bind: Measuring the strength of consumers’ emotional attachments to brands. Journal of Consumer Psychology.

[CR68] Tsai WHS, Men LR (2017). Consumer engagement with brands on social network sites: A cross-cultural comparison of China and the USA. Journal of Marketing Communications.

[CR70] Van Boven L, Gilovich T (2003). To do or to have? That is the question. Journal of Personality and Social Psychology.

[CR71] van Doorn J, Lemom KN, Mittal V, Nass S, Pich D, Pirner P, Verhoef PC (2010). Customer engagement behaviour: Theoretical foundations and reserach directions. Journal of Service Research.

[CR73] Vargo SL, Lusch RF, Lush RF, Vargo SL (2006). Service-dominant logic: What it is, what it is not, what it might be. The Service-Dominant Logic of Marketing: Dialog, Debate, and Directions.

[CR74] Venkatesh V, Bala H (2008). Technology acceptance model 3 and a research agenda on interventions. Decision Sciences.

[CR75] Venkatesh V, Davis FD (2000). A theoretical extension of the technology acceptance model: Four longitudinal field studies. Management Science.

[CR76] Venkatesh V, Thong JY, Xu X (2012). Consumer acceptance and use of information technology: Extending the unified theory of acceptance and use of technology. MIS Quarterly.

[CR77] Vroom V, Porter L, Lawler E (2015). Expectancy Theories. Organizational Behavior.

[CR78] Vuzix. 2021. Vuzix Blog. Amazon Alexa for Smart Glasses on the Vuzix Blade. Retrieved September 30, 2021, from https://www.vuzix.com/Blog/amazon-alexa-for-vuzix-blade-smart-glasses

[CR79] Whelan S, Wohlfeil M (2006). Communicating brands through engagement with ‘lived’ experiences. Journal of Brand Management.

